# Researchers in rheumatology should avoid categorization of continuous predictor variables

**DOI:** 10.1186/s12874-023-01926-4

**Published:** 2023-04-26

**Authors:** Zubeyir Salis, Blanca Gallego, Amanda Sainsbury

**Affiliations:** 1 The University of New South Wales, Centre for Big Data Research in Health, Kensington, NSW Australia; 2 School of Human Sciences, The University of Western Australia, Crawley, Perth, WA 6009 Australia

**Keywords:** Categorization 1, Dichotomization 2, Predictor Variable 3, Covariate 4

## Abstract

**Background:**

Rheumatology researchers often categorize continuous predictor variables. We aimed to show how this practice may alter results from observational studies in rheumatology.

**Methods:**

We conducted and compared the results of two analyses of the association between our predictor variable (percentage change in body mass index [BMI] from baseline to four years) and two outcome variable domains of structure and pain in knee and hip osteoarthritis. These two outcome variable domains covered 26 different outcomes for knee and hip combined. In the first analysis (categorical analysis), percentage change in BMI was categorized as ≥ 5% decrease in BMI, < 5% change in BMI, and ≥ 5% increase in BMI, while in the second analysis (continuous analysis), it was left as a continuous variable. In both analyses (categorical and continuous), we used generalized estimating equations with a logistic link function to investigate the association between the percentage change in BMI and the outcomes.

**Results:**

For eight of the 26 investigated outcomes (31%), the results from the categorical analyses were different from the results from the continuous analyses. These differences were of three types: 1) for six of these eight outcomes, while the continuous analyses revealed associations in both directions (i.e., a decrease in BMI had one effect, while an increase in BMI had the opposite effect), the categorical analyses showed associations only in one direction of BMI change, not both; 2) for another one of these eight outcomes, the categorical analyses suggested an association with change in BMI, while this association was not shown in the continuous analyses (this is potentially a false positive association); 3) for the last of the eight outcomes, the continuous analyses suggested an association of change in BMI, while this association was not shown in the categorical analyses (this is potentially a false negative association).

**Conclusions:**

Categorization of continuous predictor variables alters the results of analyses and could lead to different conclusions; therefore, researchers in rheumatology should avoid it.

**Supplementary Information:**

The online version contains supplementary material available at 10.1186/s12874-023-01926-4.

## Background

Epidemiological research can suggest potential risk factors and strategies to prevent, delay or reverse osteoarthritis and other rheumatic diseases. In epidemiological research in osteoarthritis and other rheumatic diseases, it is common practice to categorize a continuous variable that is a predictor of an outcome (the ‘predictor variable’), as evident in studies published in the past two years (2020 to 2022 [1–7]). These studies categorized continuous predictor variables such as: change in weight [8]; change in body mass index (BMI) [2]; risk score for mortality [3]; age [3]; appendicular lean mass index [4]; fat mass index [4]; disease activity score [9]; years of use of analgesics [5]; patient global visual analogue scale assessment of disease activity [10]; the Stanford Health Assessment Questionnaire-Disability Index [10]; glucocorticoid drug dosage [10]; swollen joint count [6]; tender joint count [6]; and alcohol unit consumption per week [7]. To further exemplify how common the practice of categorization of continuous predictor variables is in rheumatology research, we surveyed articles published in 2021 (the year before commencing this current study) in the journal titled *Arthritis Care & Research*. *Arthritis Care & Research* is an official journal of the *American College of Rheumatology*, which is a leading professional organization in rheumatology. In our survey, we only included articles reporting observational studies and randomized trials. Our survey revealed that 49% (101 [11–111] of 208 [11–218]) of those articles categorized the continuous predictor variables that were used in their primary analysis. Categorizing continuous variables is not specific to research in osteoarthritis and other rheumatic diseases. Indeed, a review of 58 articles published in the two months of December 2007 and January 2008 in ten journals (five epidemiological and five general medicine) found that 86% of these articles categorized the primary predictor variable [219]. A more recent review of 23 observational studies published between April and June 2015 found that 61% categorized continuous predictor variables [220].

Although it is widely used, categorization of continuous predictor variables [221–227] or continuous outcome variables [228–234] is not recommended in research because of several issues: distortion of associations [235]; loss of power and precision [236, 237]; increased probability of biased estimates [237, 238]; type I errors (false positives) [239]; type II errors (false negatives) [240]; and inflated effect sizes (odds ratio) [223, 241].

The common practice of categorizing continuous predictor variables in epidemiological research in osteoarthritis and other rheumatic diseases despite the drawbacks mentioned above may be due to the need for clarity on how this practice changes the results and conclusions. Therefore, our primary aim in this study was to investigate the extent to which the categorization of continuous predictor variables changes findings in epidemiological rheumatology research. For this study, we will use as our example the percentage change in BMI as a predictor variable, with the percentage change in BMI treated either as 1) a categorical variable or 2) a continuous variable, and the two outcome variable domains of structure and pain in knee and hip osteoarthritis.

## Methods

We revisited a study by Joseph et al. [2] that investigated the association between percentage change in BMI over four years and the two outcome variable domains of structure and pain in knee and hip osteoarthritis using data from the Osteoarthritis Initiative (OAI) study. The authors treated the percentage change in BMI as a categorical variable. From their results, they suggested that while a decrease of 5% or more in BMI may protect against overall structural changes in the knee (as assessed by radiography) and may decrease pain in the knee over four years, an increase of 5% or more in BMI may exacerbate medial joint space narrowing (JSN) in the knee and the development of pain in the knee over four years [2]. There was no association of the percentage change in BMI – when treated as a categorical variable – with any outcomes of hip osteoarthritis [2].

### Data

We used data from the OAI study [242]. OAI data is openly available to researchers for scientific and educational purposes. The OAI is a multi-center longitudinal study that collected data over four years from a total of 4796 adults (45 to 79 years of age) with or at risk of clinically significant knee osteoarthritis. The local institutional review boards of the OAI centers reviewed and approved the informed consent documentation and ethics approval.

### Exposures

Our predictor variable was the percentage change in BMI between baseline and four years, calculated as follows [2]. We fitted a simple linear regression line for each participant to estimate their annual rate of change in BMI, based on their data for BMI at baseline and other available time points. We then multiplied the slope of this regression line by 4 to estimate the absolute change in BMI over four years. The percentage change in BMI for each individual was then calculated as the absolute change in BMI over four years divided by the baseline BMI of that individual [2]. Fitting a simple linear regression line for each participant allowed us to estimate the change in BMI in cases of missing data, by using all available data points.

For the ‘categorical analysis’, we created 3 weight change groups: ≥ 5% decrease in BMI, < 5% change in BMI (i.e., stable BMI, the reference category), and ≥ 5% increase in BMI between baseline and four years. As opposed to the study by Joseph et al. [2], we did not exclude participants who showed a modest change in BMI (3–5%); and we defined the “stable BMI” category (which was the reference category) as those individuals who exhibited a change in BMI of less than 5%, whereas it was defined by Joseph et al. [2] as a change in BMI of less than 3%. By including participants that exhibited modest change in BMI, we have increased our sample size by 26.2%, and have therefore increased statistical power in our study [243, 244]. We used a 5% weight change threshold because prior studies suggest that this degree of weight change is clinically relevant [2].

For the ‘continuous analysis’, we treated the percentage change in BMI between baseline and four years as the continuous variable that it is.

### Outcomes

Our two outcome variable domains of structure and pain of knee and hip osteoarthritis covered a total of 26 outcomes (18 in the structure and 8 in the pain outcome variable domains). The definitions of these 26 outcomes are detailed in the supplementary file. These outcomes were defined based on the definitions in the study by Joseph et al. [2].

The 18 outcomes that were in the outcome variable domains of structure were as follows: eight outcomes related to the progression of knee osteoarthritis as assessed by radiography at four years’ follow up; eight outcomes related to the progression of hip osteoarthritis, also assessed by radiography at four years’ follow up; one outcome for the incidence of total knee replacement (TKR) over four years; and one outcome for the incidence of total hip replacement (THR) over four years. For our eight outcomes related to the progression of knee osteoarthritis, we separately investigated the overall structure of the knee joint, and the following seven individual structural features (ISFs) of the knee joint: 1) joint space narrowing (JSN) in the medial or lateral compartment; 2) JSN in the medial compartment; 3) JSN in the lateral compartment; 4) osteophytes on the medial tibial surface; 5) osteophytes on the lateral tibial surface; 6) osteophytes on the medial femoral surface; and 7) osteophytes on the lateral femoral surface. For our eight outcomes for the progression of hip osteoarthritis, we also separately investigated the overall structure of the hip joint, and the following seven ISFs of the hip joint: 1) JSN in the medial or lateral compartment; 2) JSN in the medial compartment; 3) JSN in the lateral compartment; 4) osteophytes on the superior acetabular surface; 5) osteophytes on the superior inferior surface; 6) osteophytes on the superior femoral surface; and 7) osteophytes on the inferior femoral surface.

In the outcome variable domain of pain, two types of pain were investigated for the knee and hip: “frequent pain” and “any pain”. For frequent pain in the knee and hip, we used the following 4 outcomes in the analyses: 1) development of frequent pain in the knee 2) development of frequent pain in the hip; 3) resolution of frequent pain in the knee; and 4) resolution of frequent pain in the hip, by four years’ follow up. For any pain in the knee and hip, we used the following 4 outcomes in the analyses: 1) development of any pain in the knee 2) development of any pain in the hip; 3) resolution of any pain in the knee; and 4) resolution of any pain in the hip, by four years’ follow up.

### Participant selection

**Fig. 1:**
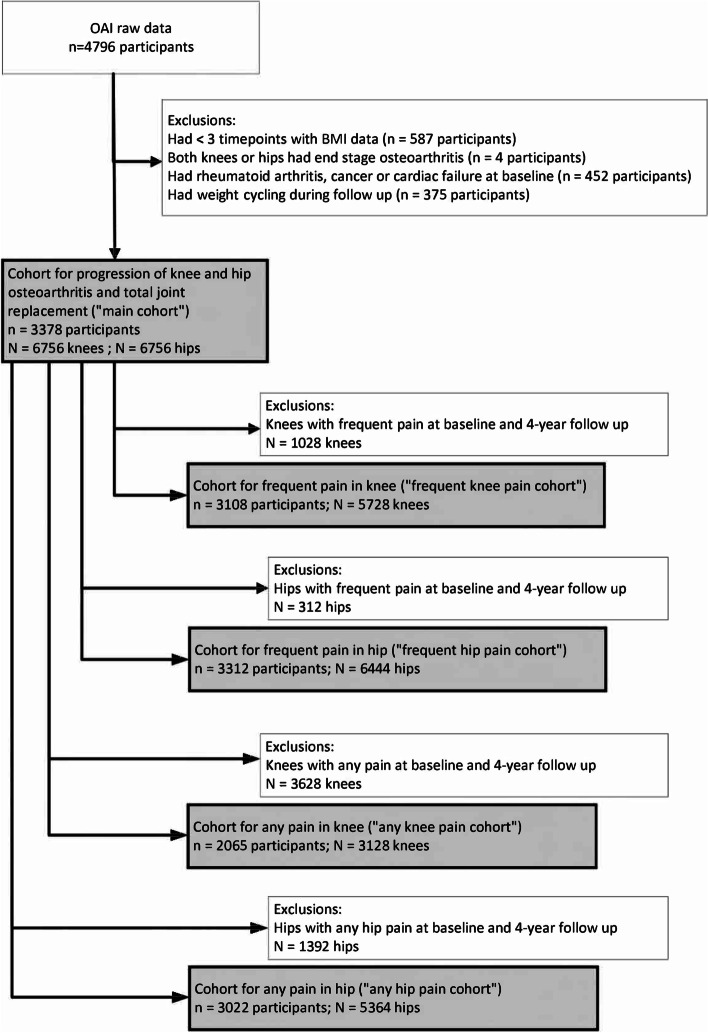
Selection of participants for each cohort. OAI: Osteoarthritis Initiative; BMI, body mass index

We applied exclusion criteria for participant selection as per the study by Joseph et al. [2]. Firstly, we excluded participants that had BMI data at less than three of the five available timepoints (Fig. 1). This was due to needing a minimum of three timepoints with BMI data to determine weight cycling (to be explained below) from BMI fluctuation. Secondly, we excluded participants who had end stage osteoarthritis of knees or hips at baseline (Fig. 1). End stage osteoarthritis of knees was defined as having a Kellgren Lawrence (KL) grade of 4 (the highest possible KL grade) in *both* knees. End stage osteoarthritis of hips was defined as having JSN that had an Osteoarthritis Research Society International (OARSI) grade of 3 (the highest possible OARSI grade) in *both* hips, in any of the two sides of the hip (i.e., lateral or medial). Exclusion of these participants was done to avoid any possible confounding effect of their data on the study results due to their potentially reduced mobility and / or reduced ability to exercise. Additionally, there is no way to assess further change in the structure of the knee or hip joints as assessed radiographically once a participant has reached end-stage osteoarthritis. Thirdly, we also excluded participants with rheumatoid arthritis, cancer, or cardiac failure at baseline, as these conditions may cause pathological weight change, which in turn can impact change in BMI (Fig. 1). Fourthly, using BMI fluctuation information, we excluded participants who had ‘weight cycling’ during follow up. Weight cycling refers to a repetitive pattern of weight loss and regain [245]. We excluded participants with weight cycling as they would not completely be classified in the weight loss or weight gain categories. Moreover, weight cycling is associated with increased progression of structural defects in osteoarthritis, regardless of whether there is net weight gain or net weight loss [246]. Weight cycling was defined based on BMI fluctuation. BMI fluctuation was calculated as the root mean square error (RMSE) of the regression line of BMI over time that was calculated for each individual [2]. The participants with a RMSE value in the top 10% of all RMSE values were determined as having weight cycling and were thus excluded [2] (Fig. 1).

With the application of these four selection criteria, the ‘main cohort’ was created, which was used for investigating the 18 outcomes in the outcome variable domain of structure for the progression of knee and hip osteoarthritis and the incidence of TKR and THR. Further, we created four additional sub-cohorts (the ‘frequent knee pain cohort’, ‘frequent hip pain cohort’, ‘any knee pain cohort’, and ‘any hip pain cohort’) which was used for investigating the 8 outcomes in the outcome variable domain of pain (Fig. 1). The 4 outcomes for frequent knee and hip pain were investigated in the ‘frequent knee pain cohort’ and ‘frequent hip pain cohort’, respectively. The 4 outcomes for any knee and hip pain were investigated in the ‘any knee pain cohort’ and ‘any hip pain cohort’, respectively.

### Statistical analyses

We used STATA/BE 17.0 for our analyses. We set our threshold for statistical significance as a two-tailed *P-*value of less than 0.05, as in the study by Joseph et al. [2]. We have not adjusted the significance level for multiple testing (e.g., Bonferroni adjustment).

We investigated the association between the percentage change in BMI (treated categorically and continuously) and the outcomes described above using generalized estimating equations with a logistic link function [247], sometimes referred to as logistic regression with clustering within individuals. In this case, the clustering is of the left and right knee or hip. This approach takes into account the within-person correlation between the two knees or hips and allows for a more accurate estimation of any association between the exposure and outcome. All analyses were adjusted for the following variables: age, sex, and baseline BMI.

For the continuous analysis, we first determined whether the percentage change in BMI had a linear relationship with each of our outcomes using the Box-Tidwell method. In this method, an interaction between the percentage change in BMI and its natural logarithmic value is added to the model. A significant interaction indicated a nonlinearity between the percentage change in BMI and the outcome variable [248]. While our statistical analysis suggested that 25 of the 26 outcomes had an apparent linear relationship with the percentage change in BMI, there may be some degree of uncertainty regarding the existence of these relationships, as the inference of linear relationships was based on the results of statistical tests. The remaining outcome, overall structural defects in knee osteoarthritis, did not show any apparent linear relationship with change in BMI. For those 25 outcomes that did have a linear relationship, we fitted a line from the available continuous range of BMI change where the relationship with the outcome variable is linear on the log odds ratio scale, then estimated the effect sizes (odds ratios) from that line. We reported the point estimates of a 5% decrease and a 5% increase in BMI. For the one outcome that did not show any apparent linear association with the percentage change in BMI (i.e., overall structural defects in knee osteoarthritis), we used the statistical method of piecewise linear spline regression. In this method, we divided the data into three separate segments: a decrease of ≥ 5% in BMI; a change of < 5% in BMI; and an increase of ≥ 5% in BMI. In each of the three segments, the change in BMI was linear, but with each segment potentially having a different effect size. We calculated effect sizes from two of these 3 separate segments; one effect size from the segment of decrease of 5% or more in BMI; the other effect size from the segment of increase of 5% or more in BMI. We used these two segments to calculate the point estimates of the effect sizes at a 5% decrease in BMI and a 5% increase in BMI.

### Sensitivity analyses

In our primary analyses (where we investigated the association between the percent change in BMI and 26 outcomes from the outcome variable domains of structure and pain in knee and hip osteoarthritis), the estimates were calculated using a 5% change in BMI in the categorical and continuous analyses. We performed sensitivity analyses to assess if our conclusions from the results that were obtained in our primary analyses would still hold for different percentage changes in BMI. For that, we performed sensitivity analyses by repeating the primary analyses but this time instead of 5%, using a 3% change in BMI categories (i.e., ≥ 3% decrease in BMI, < 3% change in BMI, and ≥ 3% increase in BMI) and a 10% change in BMI categories (i.e., ≥ 10% decrease in BMI, < 10% change in BMI, and ≥ 10% increase in BMI).

## Results

### Participant characteristics

There were 3378 participants with 6756 knees and 6756 hips in the main cohort (the cohort in which we investigated the 18 outcomes in the outcome variable domain of structure). There were 3108 participants with 5728 knees in the frequent knee pain cohort, 3312 participants with 6644 hips in the frequent hip pain cohort, 2065 participants with 3128 knees in the any knee pain cohort, and 3022 participants with 5364 hips in the any hip pain cohort (Fig. 1).

Table 1 shows characteristics of the participants included in each of the five cohorts in this study. The mean age of participants in each cohort was similar, ranging from 61.1 (standard deviation [SD] 9.2) to 61.9 (SD 9.3) years. The percentage of female participants was higher than that of male participants in each cohort, ranging from 55.9 to 57.4%. The mean BMI of participants in each cohort was also similar, ranging from 27.7 (SD 4.4) to 28.1 (SD 4.6) kg/m^2^.

**Table 1:** Baseline characteristics of participants in each cohort

	**Main cohort (The cohort for progression of knee and hip osteoarthritis and total joint replacement)**	**Frequent knee pain cohort**	**Frequent hip pain cohort**	**Any knee pain cohort**	**Any hip pain cohort**
**No of participant s**	***n*** ** = 3378**	***n*** ** = 3108**	***n*** ** = 3312**	***n*** ** = 2065**	***n*** ** = 3022**
Age, years	61.1 ± 9.2	61.2 ± 9.2	61.1 ± 9.2	61.9 ± 9.3	61.2 ± 9.3
Sex					
Male	1439 (42.6)	1343 (43.2)	1416 (42.7)	906 (43.9)	1333 (44.1)
Female	1939 (57.4)	1765 (56.8)	1896 (57.3)	1159 (56.1)	1689 (55.9)
BMI, kg/m^2^	28.1 ± 4.6	28.0 ± 4.5	28.1 ± 4.6	27.7 ± 4.4	28.1 ± 4.6
Missing	0	0	0	0	0

Data are presented as mean ± standard deviation or count (percentage)

*BMI* Body Mass Index

**Fig. 2:**
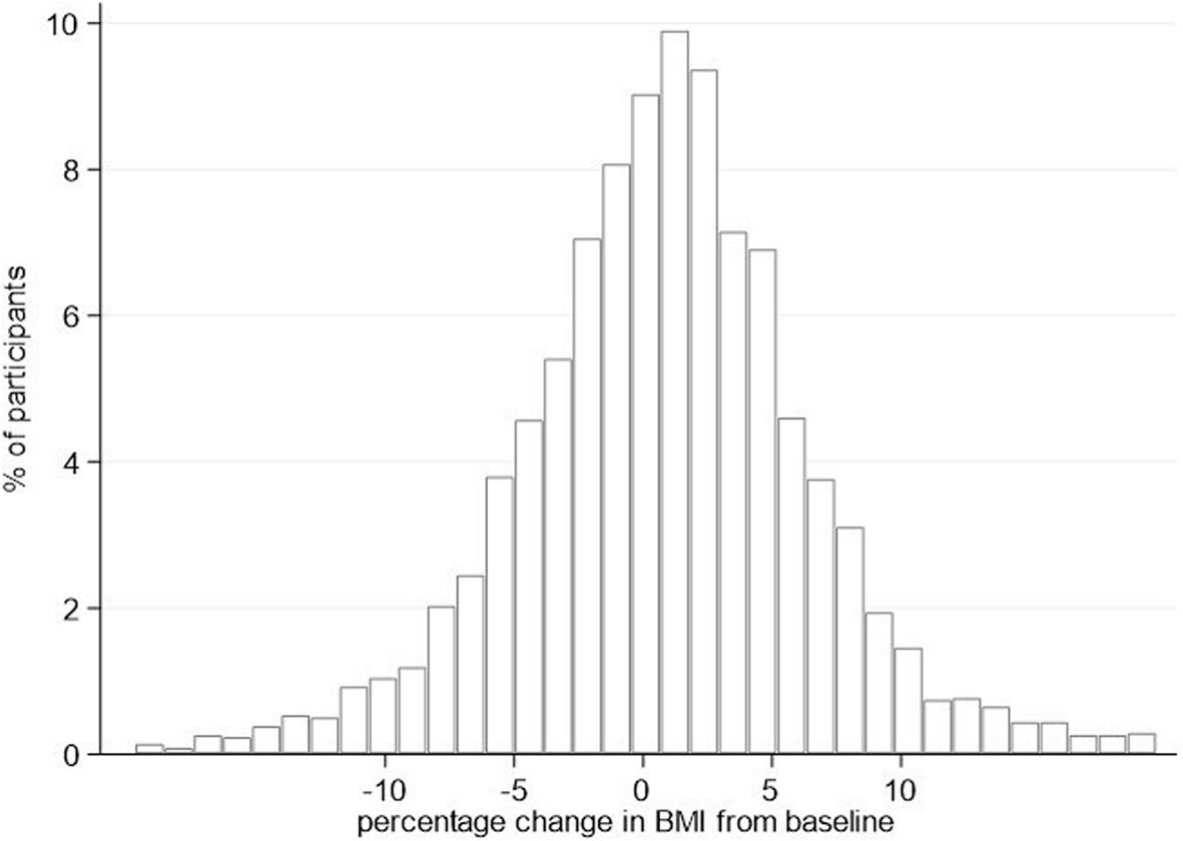
Distributions of participants by percentage change in body mass index (BMI) from baseline. BMI: Body Mass Index

Figure 2 shows the distribution of participants by the percentage change in BMI from baseline to four years’ follow up in the main cohort. Of the 3378 participants in this cohort, there were 469 (13.9%) that had a decrease in BMI of 5% or more, 2223 (65.8%) that had a stable BMI (change of less than 5%), and 686 (20.3%) that had an increase in BMI of 5% or more. The distribution of percentage change in BMI was similar in all the other four sub-cohorts (data not shown).

### Incidence of outcomes

The incidence count of outcomes in the five cohorts can be found in Tables S1 to S5 in the Supplementary file. In comparison to knee, the numbers for the incidence of outcomes for hip were generally lower, with the exception of development and resolution of any hip pain (12.7% versus 14.2% for the development of pain, and 13.6% versus 15.4% for the resolution of pain of the knee and hip, respectively).

### Association between percentage change in BMI and outcomes

Table 2 shows the results from our two analyses (categorical and continuous) for the associations of a 5% change in BMI with the 26 outcomes. Of the 26 outcomes investigated, 18 (69%) showed the same result in both the categorical and continual analyses. Of these 18 outcomes, 17 showed no association with the percentage change in BMI when treated either categorically or continuously. These 17 outcomes were: (for the knee) progression in lateral JSN; progression in medial tibial osteophytes; progression in lateral tibial osteophytes; progression in lateral femoral osteophytes; and incidence of TKR; (and for the hip) progression in overall structural defects in hip osteoarthritis; progression in medial or lateral JSN; progression in medial JSN; progression in lateral JSN; progression in superior acetabular osteophytes; progression in superior femoral osteophytes; progression in inferior femoral osteophytes; development of frequent pain in hip; resolution of frequent pain in hip; development of any pain in hip; resolution of any pain in hip; and incidence of THR (Table 2). The remaining one of these 18 outcomes (i.e., progression in overall structural defects in knee osteoarthritis) showed an association with a decrease in BMI but not with an increase in BMI in both the continuous and the categorical analysis.

**Table 2:** The associations of the percentage change in BMI with outcomes of knee and hip osteoarthritis, when percentage change in BMI is either treated as a categorical variable (in the categorical analysis, left), or as a continuous variable (in the continuous analysis, right)

Outcomes	Categorical analysis (The percentage change in BMI is treated as a categorical variable)		Continuous analysis (The percentage change in BMI is treated as a continuous variable)	
	5% or more decrease in BMI^a^	5% or more increase in BMI^a^	5% decrease in BMI	5% increase in BMI
	Odds ratio (95% confidence interval) *P*-value			
Knee				
**Knee structure**				
Progression in overall structural defects	0.68 (0.51 0.91) **0.010**	0.95 (0.76 1.20) 0.680	0.93 (0.87 0.99) **0.025**	1.02 (0.98 1.06) 0.300
Progression in medial or lateral JSN	0.58 (0.42 0.81) **0.001**	1.01 (0.79 1.28) 0.958	0.90 (0.83 0.98) **0.011**	1.11 (1.02 1.20) **0.011**
Progression in medial JSN	0.53 (0.35 0.79) **0.002**	1.10 (0.84 1.45) 0.497	0.86 (0.78 0.95) **0.002**	1.16 (1.06 1.28) **0.002**
Progression in lateral JSN	0.74 (0.43 1.27) 0.268	0.79 (0.49 1.26) 0.314	1.01 (0.87 1.16) 0.907	0.99 (0.86 1.14) 0.907
Progression in medial tibia osteophytes	0.89 (0.67 1.18) 0.415	1.19 (0.94 1.51) 0.143	0.94 (0.87 1.02) 0.115	1.06 (0.98 1.15) 0.115
Progression in lateral tibia osteophytes	1.02 (0.73 1.43) 0.887	0.96 (0.71 1.30) 0.792	1.04 (0.95 1.15) 0.415	0.96 (0.87 1.06) 0.415
Progression in medial femoral osteophytes	0.66 (0.49 0.91) **0.010**	1.22 (0.96 1.55) 0.098	0.85 (0.79 0.93) ** < 0.001**	1.17 (1.08 1.27) ** < 0.001**
Progression in lateral femoral osteophytes	0.97 (0.69 1.37) 0.880	0.97 (0.72 1.30) 0.821	0.98 (0.89 1.08) 0.672	1.02 (0.93 1.12) 0.672
Incidence of total knee replacement	0.94 (0.53 1.64) 0.818	1.15 (0.57 2.35) 0.692	0.99 (0.85 1.16) 0.916	1.01 (0.86 1.18) 0.916
**Pain in knee**				
Development of frequent pain	0.98 (0.76 1.26) 0.848	1.41 (1.15 1.73) **0.001**	0.90 (0.84 0.96) **0.003**	1.11 (1.04 1.19) **0.003**
Development of any pain	0.89 (0.68 1.16) 0.383	1.11 (0.88 1.40) 0.365	0.92 (0.85 0.99) **0.020**	1.09 (1.01 1.17) **0.020**
Resolution of frequent pain	1.31 (1.04 1.64) **0.021**	0.93 (0.75 1.14) 0.485	1.11 (1.04 1.18) **0.003**	0.90 (0.85 0.97) **0.003**
Resolution of any pain	1.41 (1.10 1.82) **0.007**	1.00 (0.80 1.27) 0.968	1.10 (1.02 1.18) **0.011**	0.91 (0.85 0.98) **0.011**
Hip				
**Hip structure**				
Progression in overall structural defects	0.94 (0.60 1.48) 0.798	1.21 (0.84 1.75) 0.306	0.96 (0.85 1.08) 0.527	1.04 (0.92 1.17) 0.527
Progression in medial or lateral JSN	1.20 (0.84 1.72) 0.318	1.18 (0.85 1.65) 0.324	0.95 (0.85 1.06) 0.343	1.06 (0.94 1.18) 0.343
Progression in medial JSN	1.28 (0.84 1.93) 0.248	1.15 (0.77 1.71) 0.495	0.96 (0.84 1.10) 0.549	1.04 (0.91 1.19) 0.549
Progression in lateral JSN	1.03 (0.61 1.74) 0.902	1.17 (0.74 1.85) 0.502	0.98 (0.84 1.16) 0.852	1.02 (0.87 1.19) 0.852
Progression in superior acetabular osteophytes	1.80 (0.97 3.34) 0.062	1.62 (0.91 2.89) 0.099	1.09 (0.89 1.34) 0.392	0.91 (0.74 1.12) 0.392
Progression in inferior acetabular osteophytes progression ~	2.70 (1.14 6.41) **0.024**	1.75 (0.71 4.32) 0.225	1.18 (0.86 1.62) 0.307	0.85 (0.62 1.16) 0.307
Progression in superior femoral osteophytes	1.05 (0.64 1.74) 0.841	1.05 (0.66 1.67) 0.826	1.04 (0.89 1.21) 0.658	0.97 (0.83 1.13) 0.658
Progression in inferior femoral osteophytes	0.74 (0.30 1.81) 0.512	0.78 (0.34 1.83) 0.574	0.99 (0.77 1.28) 0.950	1.01 (0.78 1.30) 0.950
Incidence of total hip replacement	1.15 (0.57 2.35) 0.692	1.09 (0.55 2.17) 0.806	1.01 (0.82 1.24) 0.947	0.99 (0.81 1.22) 0.947
**Pain in hip**				
Development of frequent pain	0.95 (0.71 1.28) 0.754	1.11 (0.88 1.41) 0.387	0.95 (0.87 1.02) 0.160	1.06 (0.98 1.15) 0.160
Development of any pain	0.86 (0.67 1.10) 0.227	0.94 (0.77 1.16) 0.582	0.99 (0.93 1.06) 0.779	1.01 (0.94 1.08) 0.779
Resolution of frequent pain	1.06 (0.79 1.42) 0.698	0.87 (0.67 1.14) 0.314	1.03 (0.95 1.12) 0.457	0.97 (0.89 1.05) 0.457
Resolution of any pain	1.05 (0.83 1.33) 0.697	1.14 (0.93 1.40) 0.201	0.98 (0.92 1.05) 0.541	1.02 (0.96 1.09) 0.541

*JSN* Joint Space Narrowing. Adjusted for age, gender and Body Mass Index (BMI) at baseline

^a^Compared to < 5% change in BMI (i.e., stable BMI)

Of the 26 outcomes investigated, the remaining eight outcomes (31%) showed association with a 5% change in BMI in either the categorical or the continuous analysis, but not in both analyses (Table 2). These eight outcomes were: (for the knee) 1) progression in medial or lateral JSN; 2) progression in medial JSN; 3) progression in medial femoral osteophytes; 4) development of frequent pain in knee; 5) resolution of frequent pain in knee; 6) development of any pain in knee; 7) resolution of any pain in knee; and (for the hip) 8) inferior acetabular osteophyte progression (Table 2).

Although these eight outcomes were associated with the percentage change in BMI when treated categorically or continuously, there were three types of differences between the associations in the two analyses. These differences will be explained below in points a, b, and c.

- *Outcomes showed associations with percentage change in BMI only in one direction in the categorical analyses but in both directions in the continuous analyses*
  Six of these eight outcomes were positively associated with the percentage change in BMI (i.e., both increase and decrease in BMI) when BMI was treated as a continuous variable. However, in the categorical analysis, these six outcomes had an association with either an increase in BMI or a decrease in BMI, but not both. In the categorical analyses, five of these six outcomes were only associated with a decrease in BMI but not an increase in BMI. These five outcomes (all for knee) were: 1) progression in medial or lateral JSN; 2) progression in medial JSN; 3) progression in medial femoral osteophytes; 4) resolution of frequent pain in knee; and 5) resolution of any pain in knee. The remaining one outcome (development of frequent pain in knee) showed an association with an increase in BMI but not with a decrease in BMI in the categorical analysis (Table 2).
- *Outcomes showed associations with percentage change in BMI in the categorical analysis but not in the continuous analysis (possible false positive)*
  When BMI was treated as a categorical variable, one of these eight outcomes, namely progression in inferior acetabular osteophytes in the hip, showed an association with a decrease in BMI (but not an increase in BMI). This may be a false positive, because 1) the outcome showed no significant association with the percentage change in BMI when percentage change in BMI was treated as a continuous variable (Table 2); 2) there was no other significant association for the any of the 8 outcomes for progression of hip osteoarthritis assessed by radiography in the categorical analysis (Table 2); and 3) acetabular osteophytes are not a reliable measure for the progression of hip osteoarthritis [249, 250] as it is difficult to distinguish them from normal anatomy [250].
- *Outcomes showed associations with percentage change in BMI in the continuous analysis but not in the categorical analysis (possible false negative)*
  When BMI was treated as a categorical variable, one of these eight outcomes, namely the development of any pain in the knee, showed no association with the percentage change in BMI (either a decrease in BMI or an increase in BMI). This may be a false negative, because 1) the outcome showed an association with the percentage change in BMI when percentage change in BMI was treated as a continuous variable (Table 2); 2) all the other 3 of 4 outcomes for knee pain showed an association either with a decrease or increase in BMI in the categorical analysis, suggesting likelihood of an association; and 3) other studies showed an association of change in BMI with the development of knee pain due to osteoarthritis [251, 252].

### Sensitivity analyses

The results from the sensitivity analyses using 3% and 10% change in BMI (decrease or increase) showed that eight and seven of the 26 outcomes investigated, respectively, differed in the categorical compared to the continuous analysis, showing all of the three different types of differences that were shown in our primary analyses (i.e., using 5% change in BMI) (Table S6).

## Discussion

This study in osteoarthritis showed that categorizing the continuous predictor variable in the analysis (in this example, the percentage change in BMI) could influence the results in three ways. The first of these three ways was that statistically significant associations were found in one direction when percentage change in BMI was treated categorically. In contrast, they were found in both directions when percentage change in BMI was left as a continuous variable. The second of these three ways was by showing statistically significant associations that are non-existent when the variable is left as a continuous variable (possible false positives) [240]. Specifically, in our categorical analysis, the outcome of progression in inferior acetabular osteophytes in the hip was associated with a decrease in BMI. In contrast, it was not associated with either a decrease or increase in BMI in the continuous analysis (Table 2). The third way was that the analyses with categorized continuous variables might mask statistically significant associations when the variable is left as a continuous variable (possible false negatives) [239]. Specifically, in our categorical analysis, the outcome of the development of any pain in knee was not associated with a decrease nor an increase in BMI. In contrast, it was associated with both a decrease and an increase in BMI in the continuous analysis (Table 2). Further, our sensitivity analyses using 3% and 10% changes in BMI delivered the same conclusions as our primary analyses (that used a 5% change in BMI), showing that these three issues with categorization of continuous variables are independent of these different thresholds of the continuous variable (percentage change in BMI).

Of these three ways that results differed depending on whether the predictor variable was treated as a continuous or a categorical variable, the first one was a major problem as the conclusions drawn from the continuous and categorical analyses results would be different. From the continuous analyses in this study, we would conclude a beneficial association between a decrease in BMI and a harmful association of an increase in BMI for structural changes and pain in knee osteoarthritis over four years, as the effect of percentage change in BMI was shown in both directions (decrease and increase). However, from the categorical analysis in this study, we would have concluded that a decrease in BMI is associated with beneficial effects for knee structure and pain in osteoarthritis but that an increase in BMI is not associated with harmful effects. The conclusion about lack of association between an increase in BMI from the categorical analyses conflicts with the conclusion from the continuous analyses, as well as from previous research showing that weight gain is associated with harmful effects of structural changes and pain in knee osteoarthritis while weight loss is associated with beneficial effects [249, 253–256]. It is difficult to reconcile that a decrease in BMI is associated with one effect, whereas an increase in BMI is not associated with the opposite effect. This difficulty in the reconciliation of the results from the analysis using categorized continuous variables can also be seen in the study by Joseph et al.[2] which we revisited. That study, which used categorized percentage change in BMI, showed the association of either a decrease in BMI or an increase in BMI with outcomes of structural changes and pain in knee osteoarthritis, but not for both a decrease and an increase in BMI for any outcome.

We acknowledge the limitations in our study. There were 276 (5.8%) of the 4,796 participants with missing data for whom we could not estimate their BMI change. Therefore, we cannot exclude the possibility that missing data could have resulted in bias in our estimates. Additionally, it is important to note that our study only investigated the impact of categorizing one predictor variable (percentage change in BMI) on several outcomes in one specific population (OAI). Therefore, our results cannot be generalized to all situations in which researchers categorize variables in rheumatology. Categorization of continuous predictor variables can be useful in rheumatology research when there is strong prior knowledge or established cut-offs for a particular variable, such as disease activity scores, categorization of antibody titers (anti-citrullinated protein antibody, ACPA positive/negative), or achieving remission or not (yes/no). In such cases, categorization can aid in simplifying the analysis and interpretation of the results.

## Conclusions

In conclusion, our study demonstrated that categorizing continuous predictor variables in rheumatology may result in associations being shown in only one direction, and could also lead to possible false positive and possible false negative associations, which may lead to erroneous conclusions. We suggest that researchers in rheumatology, including clinicians and peer reviewers, consider the potential drawbacks of categorizing continuous predictor variables and prioritize the use of continuous variables.

## Supplementary Information

**Additional file 1: Definitions of outcomes. Table S1.** Incidence of outcomes in main cohort (the cohort for investigating the progression of knee and hip osteoarthritis and the incidence of total joint replacement). **Table S2.** Incidence of outcomes in frequent knee pain cohort. **Table S3.** Incidence of outcomes in frequent hip pain cohort. **Table S4.** Incidence of outcomes in any knee pain cohort. **Table S5.** Incidence of outcomes in any hip pain cohort. **Table S6.** The associations of change in BMI change with outcomes, as treating change in BMI categorical (using 3, 5 and 10% weight change categories) and continuous variable.

## Data Availability

OAI data is openly available to researchers for scientific and educational purposes in the following website https://nda.nih.gov/oai/. The STATA code can be obtained from the authors upon request.

## Abbreviations

BMI: Body Mass Index
CI: Confidence Interval
ISF: Individual Structural Feature
JSN: Joint Space Narrowing
KL: Kellgren Lawrence
OAI: Osteoarthritis Initiative
OARSI: Osteoarthritis Research Society International
OR: Odds Ratio
RMSE: Root Mean Square Error
SD: Standard Deviation
THR: Total Hip Replacement
TKR: Total Knee Replacement
