# Supplementary material for: Researchers in rheumatology should avoid categorization of continuous predictor variables
Source: BMC Med Res Methodol. 2023 Apr 26;23:104. doi: 10.1186/s12874-023-01926-4 (PMC10134601; doi:10.1186/s12874-023-01926-4)
Supplement: Supplementary file 1 — Additional file 1: Definitions of outcomes. Table S1. Incidence of outcomes in main cohort (the cohort for investigating the progression of knee and hip osteoarthritis and the incidence of total joint replacement). Table S2. Incidence of outcomes in frequent knee pain cohort. Table S3. Incidence of outcomes in frequent hip pain cohort. Table S4. Incidence of outcomes in any knee pain cohort. Table S5. Incidence of outcomes in any hip pain cohort. Table S6. The associations of change in BMI change with outcomes, as treating change in BMI categorical (using 3, 5 and 10% weight change categories) and continuous variable. [file 12874_2023_1926_MOESM1_ESM.docx]

**Supplementary File**

**Researchers in rheumatology should avoid the categorization of continuous predictor variables**

Zubeyir Salis; Blanca Gallego; Amanda Sainsbury

Contents

[**Definitions of outcomes** 2](#_Toc133169599)

[Outcomes variable domain of structure 2](#_Toc133169600)

[Outcomes variable domain of pain 4](#_Toc133169601)

[**Table S1.** Incidence of outcomes in main cohort (the cohort for investigating the progression of knee and hip osteoarthritis and the incidence of total joint replacement) (total of 18 outcomes) 6](#_Toc133169602)

[**Table S2.** Incidence of outcomes in frequent knee pain cohort (total of 2 outcomes) 9](#_Toc133169603)

[**Table S3.** Incidence of outcomes in frequent hip pain cohort (total of 2 outcomes) 10](#_Toc133169604)

[**Table S4.** Incidence of outcomes in any knee pain cohort (total of 2 outcomes) 11](#_Toc133169605)

[**Table S5.** Incidence of outcomes in any hip pain cohort (total of 2 outcomes) 12](#_Toc133169606)

[**Table S6**. The associations of change in BMI change with outcomes, as treating change in BMI categorical (using 3%, 5% and 10% weight change categories) and continuous variable. 13](#_Toc133169607)

# **Definitions of outcomes**

Our two outcome variable domains of structure and pain of knee and hip osteoarthritis covered a total of 26 outcomes. To investigate these 26 outcomes, we created five cohorts: one main cohort (‘main cohort’), and four sub-cohorts from this main cohort (‘frequent knee pain cohort’, ‘frequent hip pain cohort’, ‘any knee pain cohort’, ‘any hip pain cohort’).

## Outcomes variable domain of structure

In the outcome variable domain of structure, there were a total of 18 outcomes. These 18 outcomes were as follows: eight outcomes related to the progression of knee osteoarthritis as assessed by radiography at four years’ follow up; eight outcomes related to the progression of hip osteoarthritis, also assessed by radiography at four years’ follow up; one outcome for the incidence of total knee replacement (TKR) over four years; and one outcome for the incidence of total hip replacement (THR) over four years.

For our eight outcomes related to the progression of knee osteoarthritis, we separately investigated the overall structure of the knee joint as well as individual structural features (ISF) of the knee joint. The overall structure of the knee joint was assessed by Kellgren and Lawrence (KL) grade [[25](#_ENREF_25)]. The progression in overall structural defects of knee osteoarthritis was defined as an increase of 1 or more KL grades from baseline at the four years’ follow up. The exception to this definition was when the KL grade was 0 at baseline and 1 at the four years’ follow up, because a KL grade of 1 is considered to indicate a doubtful presence of osteoarthritis [[26](#_ENREF_26)]. In addition to the overall structure of the knee joint, we also investigated the following seven ISFs of the knee joint: 1) joint space narrowing (JSN) in the medial or lateral compartment; 2) JSN in the medial compartment; 3) JSN in the lateral compartment; 4) osteophytes on the medial tibial surface; 5) osteophytes on the lateral tibial surface; 6) osteophytes on the medial femoral surface; and 7) osteophytes on the lateral femoral surface. Each ISF was assessed by the Osteoarthritis Research Society International (OARSI) grade [[25](#_ENREF_25), [27](#_ENREF_27)]. The progression in structural defects of an ISF was defined as an increase of one or more OARSI grades from baseline at the four years’ follow up. For our eight outcomes for the progression of hip osteoarthritis, we also separately investigated the overall structure of the hip joint as well as the ISFs of the hip joint. Overall structural defects of the hip joint were assessed by the modified Croft summary grade [[2](#_ENREF_2)]. The progression in overall structural defects of hip osteoarthritis was defined as an increase of 1 or more modified Croft summary grades from baseline at the four years’ follow up. In addition to overall structure of the hip joint, we also investigated the following seven ISFs of the hip joint: 1) joint space narrowing (JSN) in the medial or lateral compartment; 2) JSN in the medial compartment; 3) JSN in the lateral compartment; 4) osteophytes on the superior acetabular surface; 5) osteophytes on the superior inferior surface; 6) osteophytes on the superior femoral surface; and 7) osteophytes on the inferior femoral surface. Each ISF was assessed by the OARSI grade [[25](#_ENREF_25), [27](#_ENREF_27)]. As in the knee joint, progression in structural defects of an ISF of the hip joint was defined as an increase of one or more OARSI grades from baseline at the four years’ follow up. For our two outcomes for the incidence of TKR and THR, we defined the incidence of TKR and THR as having a TKR and THR at any time between baseline and four years’ follow up. We identified whether or not a participant had had a TKR and THR using the variables of v99erkvsaf (for TKR) and v99erhvsaf (for THR) in the OAI dataset. We selected only TKRs and THRs that were confirmed by medical records. All of the 18 outcomes in the outcome variable domain of structure were investigated in the ‘main cohort’.

## Outcomes variable domain of pain

In the outcome variable domain of pain, there were eight outcomes. These eight outcomes were as follows: development of frequent knee pain; development of frequent hip pain; resolution of frequent knee pain; resolution of frequent hip pain; development of any knee pain; development of any hip pain; resolution of any knee pain; and resolution of any hip pain by four years’ follow up.

In the outcome variable domain of pain, two types of pain were investigated for knee and hip: “frequent pain” and “any pain”. Frequent pain in the knee or hip was defined as a participant having pain, aching or stiffness in the knee or hip, respectively, on most days of a month during the 12 months prior to when the visit was done (whether baseline or follow up). For frequent pain in the knee and hip, we used the following 4 outcomes in the analyses: 1) development of frequent pain in the knee 2) development of frequent pain in the hip; 3) resolution of frequent pain in the knee; and 4) resolution of frequent pain in the hip. Development of frequent pain in the knee and hip was defined as having the presence of frequent pain in the knee and hip at four years’ follow up when it was not present in the same knee and hip at baseline. Resolution of frequent pain in the knee and hip was defined as not having the presence of frequent pain in the knee or hip at four years’ follow up when it was present in the same knee and hip at baseline. The 4 outcomes for frequent knee and hip pain were investigated in the ‘frequent knee pain cohort’ and ‘frequent hip pain cohort’, respectively. Any pain in the knee or hip was defined as a participant having pain, aching or stiffness in the knee or hip, respectively, at any time in the 12 months prior to when the visit was done (whether baseline or follow up). For any pain in the knee and hip, we used the following 4 outcomes in the analyses: 1) development of any pain in the knee 2) development of any pain in the hip; 3) resolution of any pain in the knee; and 4) resolution of any pain in the hip. Development of any pain in the knee and hip was defined as having the presence of any pain in the knee and hip at four years’ follow up when it was not present in the same knee and hip at baseline. Resolution of any pain in the knee and hip was defined as not having the presence of any pain in the knee or hip at four years’ follow up when it was present in the same knee and hip at baseline. The 4 outcomes for any knee and hip pain were investigated in the ‘any knee pain cohort’ and ‘any hip pain cohort’, respectively.

All of the above outcomes were defined as per the definitions applied in the study by Joseph *et al* [[2](#_ENREF_2)], except for the definitions for osteophytes in the knee and hip, which were not investigated by Joseph *et al* [[2](#_ENREF_2)].

# **Table S1.** Incidence of outcomes in main cohort (the cohort for investigating the progression of knee and hip osteoarthritis and the incidence of total joint replacement) (total of 18 outcomes)

|  | **Incidence count**  **(%)** |
| --- | --- |
| **No of participant s** | ***n* = 3378** |
| **No of knee and hip joints** | **N=6756** |
| KNEE | |
| Progression in overall structural defects | 654  (11.6) |
| Progression in medial or lateral JSN | 561  (8.3) |
| Progression in medial JSN | 413  (6.1) |
| Progression in lateral JSN | 157  (2.3) |
| Progression in tibial medial osteophytes | 621  (9.2) |
| Progression in femoral medial osteophytes | 596  (8.8) |
| Progression in tibial lateral osteophytes | 392  (5.8) |
| Progression in femoral lateral osteophytes | 363  (5.4) |
| Incidence of total knee replacement | 127  (1.9) |
| HIP | |
| Progression in overall structural defects | 199  (3.0) |
| Progression in medial or lateral JSN | 316  (4.7) |
| Progression in medial JSN | 227  (3.4) |
| Progression in lateral JSN | 152  (2.3) |
| Progression in acetabular superior osteophytes | 86  (1.3) |
| Progression in acetabular inferior osteophytes | 35  (0.5) |
| Progression in femoral superior osteophytes | 152  (2.3) |
| progression in femoral inferior osteophytes | 60  (0.9) |
| Incidence of total hip replacement | 63  (0.9) |

JSN: Joint space narrowing.

# **Table S2.** Incidence of outcomes in frequent knee pain cohort (total of 2 outcomes)

|  | **Incidence count**  **(%)** |
| --- | --- |
| **No of participant s** | ***n* = 3108** |
| **No of knee joints** | **N=5728** |
| Development of frequent knee pain | 877  (13.0) |
| Resolution of frequent knee pain | 1038  (15.4) |

# **Table S3.** Incidence of outcomes in frequent hip pain cohort (total of 2 outcomes)

|  | **Incidence count**  **(%)** |
| --- | --- |
| **No of participant s** | ***n* = 3312** |
| **No of hip joints** | **N=6444** |
| Development of frequent pain in hip | 649  (9.6) |
| Resolution of frequent pain in hip | 603  (8.9) |

# **Table S4.** Incidence of outcomes in any knee pain cohort (total of 2 outcomes)

|  | **Incidence count**  **(%)** |
| --- | --- |
| **No of participants** | ***n* = 2065** |
| **No of knee joints** | **N=3128** |
| Development of any pain in knee | 856  (12.7) |
| Resolution of any pain in knee | 916  (13.6) |

# **Table S5.** Incidence of outcomes in any hip pain cohort (total of 2 outcomes)

|  | **Incidence count**  **(%)** |
| --- | --- |
| **No of participants** | **n = 3022** |
| **No of hip joints** | **N=5364** |
| Development of any pain in hip | 957  (14.2) |
| Resolution of any pain n hip | 1039  (15.4) |

# **Table S6**. The associations of change in BMI change with outcomes, as treating change in BMI categorical (using 3%, 5% and 10% weight change categories) and continuous variable.

| Outcomes | Categorical analysis  (The percentage change in BMI is treated as a categorical variable) | | Categorical analysis  (The percentage change in BMI is treated as a categorical variable) | | Categorical analysis  (The percentage change in BMI is treated as a categorical variable) | | Continuous analysis  (The percentage change in BMI is treated as a continuous variable) | | Continuous analysis  (The percentage change in BMI is treated as a continuous variable) | | Continuous analysis  (The percentage change in BMI is treated as a continuous variable) | |
| --- | --- | --- | --- | --- | --- | --- | --- | --- | --- | --- | --- | --- |
|  | 3% or more decrease in BMI**^1^** | 3% or more increase in BMI**^1^** | 5% or more decrease in BMI**^2^** | 5% or more increase in BMI**^2^** | 10% or more decrease in BMI**^3^** | 10% or more increase in BMI**^3^** | 3%  decrease in BMI | 3%  increase in BMI | 5%  decrease in BMI | 5%  increase in BMI | 10%  decrease in BMI | 10%  increase in BMI |
|  | Odds ratio (95% confidence interval)  P-value | | | | | | | | | | | |
| KNEE | | | | | | | | | | | | |
| **Knee structure** | | | | | | | | | | | | |
| Progression in overall structural defects | 0.76  (0.60 0.96)  **0.020** | 0.88  (0.71 1.07)  0.201 | 0.68  (0.51 0.91)  **0.010** | 0.95  (0.76 1.20)  0.680 | 0.65  (0.39 1.10)  0.108 | 1.05  (0.70 1.56)  0.821 | 0.93  (0.89 0.98)  **0.007** | 1.02  (0.98 1.05)  0.314 | 0.93  (0.87 0.99)  **0.025** | 1.02  (0.98 1.06)  0.300 | 0.92  (0.81 1.04)  0.171 | 1.04  (0.98 1.10)  0.197 |
| Progression in medial or lateral JSN | 0.72  (0.56 0.93)  **0.013** | 0.92  (0.74 1.14)  0.432 | 0.58  (0.42 0.81)  **0.001** | 1.01  (0.79 1.28)  0.958 | 0.63  (0.36 1.11)  0.111 | 1.04  (0.67 1.61)  0.870 | 0.94  (0.89 0.99)  **0.011** | 1.06  (1.01 1.12)  **0.011** | 0.90  (0.83 0.98)  **0.011** | 1.11  (1.02 1.20)  **0.011** | 0.81  (0.69 0.95)  **0.011** | 1.23  (1.05 1.45)  **0.011** |
| Progression in medial JSN | 0.72  (0.53 0.98)  **0.035** | 0.96  (0.74 1.24)  0.742 | 0.53  (0.35 0.79)  **0.002** | 1.10  (0.84 1.45)  0.497 | 0.54  (0.26 1.10)  0.090 | 1.32  (0.82 2.11)  0.251 | 0.91  (0.86 0.97)  **0.002** | 1.09  (1.03 1.16)  **0.002** | 0.86  (0.78 0.95)  **0.002** | 1.16  (1.06 1.28)  **0.002** | 0.74  (0.61 0.90)  **0.002** | 1.35  (1.12 1.63)  **0.002** |
| Progression in lateral JSN | 0.75  (0.47 1.18)  0.210 | 0.86  (0.58 1.28)  0.456 | 0.74  (0.43 1.27)  0.268 | 0.79  (0.49 1.26)  0.314 | 0.87  (0.35 2.11)  0.751 | 0.49  (0.17 1.44)  0.197 | 1.01  (0.92 1.10)  0.907 | 0.99  (0.91 1.08)  0.907 | 1.01  (0.87 1.16)  0.907 | 0.99  (0.86 1.14)  0.907 | 1.02  (0.76 1.36)  0.907 | 0.98  (0.74 1.31)  0.907 |
| Progression in medial tibia osteophytes | 0.81  (0.63 1.03)  0.086 | 1.10  (0.89 1.37)  0.365 | 0.89  (0.67 1.18)  0.415 | 1.19  (0.94 1.51)  0.143 | 0.99  (0.62 1.61)  0.983 | 1.51  (1.02 2.23)  **0.038** | 0.96  (0.92 1.01)  0.115 | 1.04  (0.99 1.09)  0.115 | 0.94  (0.87 1.02)  0.115 | 1.06  (0.98 1.15)  0.115 | 0.88  (0.75 1.03)  0.115 | 1.13  (0.97 1.33)  0.115 |
| Progression in lateral tibia osteophytes | 1.07  (0.80 1.43)  0.667 | 1.02  (0.77 1.33)  0.910 | 1.02  (0.73 1.43)  0.887 | 0.96  (0.71 1.30)  0.792 | 1.37  (0.80 2.33)  0.248 | 0.90  (0.53 1.53)  0.706 | 1.02  (0.97 1.08)  0.415 | 0.98  (0.92 1.03)  0.415 | 1.04  (0.95 1.15)  0.415 | 0.96  (0.87 1.06)  0.415 | 1.08  (0.89 1.31)  0.415 | 0.92  (0.76 1.12)  0.415 |
| Progression in medial femoral osteophytes | 0.69  (0.53 0.90)  **0.006** | 1.15  (0.92 1.44)  0.206 | 0.66  (0.49 0.91)  **0.010** | 1.22  (0.96 1.55)  0.098 | 0.43  (0.23 0.82)  **0.010** | 1.15  (0.76 1.74)  0.515 | 0.91  (0.87 0.95)  **<0.001** | 1.10  (1.05 1.15)  **<0.001** | 0.85  (0.79 0.93)  **<0.001** | 1.17  (1.08 1.27)  **<0.001** | 0.73  (0.62 0.86)  **<0.001** | 1.37  (1.17 1.61)  **<0.001** |
| Progression in lateral femoral osteophytes | 1.03  (0.76 1.38)  0.862 | 1.07  (0.82 1.41)  0.608 | 0.97  (0.69 1.37)  0.880 | 0.97  (0.72 1.30)  0.821 | 0.99  (0.55 1.79)  0.974 | 1.51  (0.95 2.39)  0.079 | 0.99  (0.93 1.05)  0.672 | 1.01  (0.96 1.07)  0.672 | 0.98  (0.89 1.08)  0.672 | 1.02  (0.93 1.12)  0.672 | 0.96  (0.79 1.16)  0.672 | 1.04  (0.86 1.26)  0.672 |
| Incidence of total knee replacement | 1.02  (0.62 1.67)  0.942 | 1.05  (0.66 1.66)  0.842 | 0.94  (0.53 1.64)  0.818 | 1.15  (0.57 2.35)  0.692 | 1.39  (0.64 3.00)  0.408 | 1.36  (0.60 3.04)  0.459 | 1.00  (0.91 1.09)  0.916 | 1.00  (0.92 1.10)  0.916 | 0.99  (0.85 1.16)  0.916 | 1.01  (0.86 1.18)  0.916 | 0.98  (0.72 1.34)  0.916 | 1.02  (0.75 1.38)  0.916 |
| **Pain in knee** | | | | | | | | | | | | |
| Development of frequent pain | 0.96  (0.77 1.20)  0.704 | 1.23  (1.02 1.49)  **0.034** | 0.98  (0.76 1.26)  0.848 | 1.41  (1.15 1.73)  **0.001** | 1.16  (0.76 1.78)  0.484 | 1.91  (1.37 2.65)  **<0.001** | 0.94  (0.90 0.98)  **0.003** | 1.07  (1.02 1.11)  **0.003** | 0.90  (0.84 0.96)  **0.003** | 1.11  (1.04 1.19)  **0.003** | 0.81  (0.70 0.93)  **0.003** | 1.24  (1.08 1.42)  **0.003** |
| Development of any pain | 0.95  (0.75 1.19)  0.643 | 1.16  (0.95 1.43)  0.146 | 0.89  (0.68 1.16)  0.383 | 1.11  (0.88 1.40)  0.365 | 0.90  (0.57 1.42)  0.644 | 1.62  (1.09 2.39)  **0.016** | 0.95  (0.91 0.99)  **0.020** | 1.05  (1.01 1.10)  **0.020** | 0.92  (0.85 0.99)  **0.020** | 1.09  (1.01 1.17)  **0.020** | 0.84  (0.73 0.97)  **0.020** | 1.19  (1.03 1.37)  **0.020** |
| Resolution of frequent pain | 1.18  (0.97 1.44)  0.099 | 0.89  (0.74 1.08)  0.242 | 1.31  (1.04 1.64)  **0.021** | 0.93  (0.75 1.14)  0.485 | 1.44  (0.97 2.13)  0.070 | 0.81  (0.55 1.19)  0.289 | 1.06  (1.02 1.11)  **0.003** | 0.94  (0.90 0.98)  **0.003** | 1.11  (1.04 1.18)  **0.003** | 0.90  (0.85 0.97)  **0.003** | 1.23  (1.07 1.40)  **0.003** | 0.82  (0.71 0.93)  **0.003** |
| Resolution of any pain | 1.21  (0.97 1.50)  0.097 | 0.98  (0.79 1.20)  0.808 | 1.41  (1.10 1.82)  **0.007** | 1.00  (0.80 1.27)  0.968 | 1.31  (0.85 2.02)  0.215 | 0.70  (0.45 1.08)  0.110 | 1.06  (1.01 1.10)  **0.011** | 0.95  (0.91 0.99)  **0.011** | 1.10  (1.02 1.18)  **0.011** | 0.91  (0.85 0.98)  **0.011** | 1.21  (1.04 1.39)  **0.011** | 0.83  (0.72 0.96)  **0.011** |
| HIP | | | | | | | | | | | | |
| **Hip structure** | | | | | | | | | | | | |
| Progression in overall structural defects | 1.04  (0.71 1.52)  0.828 | 1.07  (0.76 1.51)  0.694 | 0.94  (0.60 1.48)  0.798 | 1.21  (0.84 1.75)  0.306 | 0.95  (0.45 2.01)  0.888 | 1.39  (0.78 2.50)  0.266 | 0.98  (0.91 1.05)  0.527 | 1.02  (0.95 1.10)  0.527 | 0.96  (0.85 1.08)  0.527 | 1.04  (0.92 1.17)  0.527 | 0.93  (0.73 1.18)  0.527 | 1.08  (0.85 1.37)  0.527 |
| Progression in medial or lateral JSN | 1.21  (0.88 1.65)  0.238 | 1.11  (0.83 1.51)  0.478 | 1.20  (0.84 1.72)  0.318 | 1.18  (0.85 1.65)  0.324 | 0.99  (0.50 1.97)  0.983 | 1.95  (1.17 3.26)  **0.011** | 0.97  (0.90 1.04)  0.343 | 1.03  (0.97 1.11)  0.343 | 0.95  (0.85 1.06)  0.343 | 1.06  (0.94 1.18)  0.343 | 0.90  (0.71 1.12)  0.343 | 1.12  (0.84 1.40)  0.343 |
| Progression in medial JSN | 1.53  (1.06 2.20)  **0.024** | 1.15  (0.77 1.71)  0.495 | 1.28  (0.84 1.93)  0.248 | 1.15  (0.77 1.71)  0.495 | 0.95  (0.42 2.12)  0.896 | 1.73  (0.93 3.20)  0.082 | 0.98  (0.90 1.06)  0.549 | 1.02  (0.95 1.11)  0.549 | 0.96  (0.84 1.10)  0.549 | 1.04  (0.91 1.19)  0.549 | 0.92  (0.71 1.20)  0.549 | 1.08  (0.83 1.41)  0.549 |
| Progression in lateral JSN | 1.05  (0.68 1.62)  0.838 | 1.01  (0.67 1.53)  0.948 | 1.03  (0.61 1.74)  0.902 | 1.17  (0.74 1.85)  0.502 | 1.23  (0.50 3.01)  0.648 | 1.80  (0.86 3.77)  0.118 | 0.99  (0.90 1.10)  0.852 | 1.01  (0.92 1.11)  0.852 | 0.98  (0.84 1.16)  0.852 | 1.02  (0.87 1.19)  0.852 | 0.97  (0.71 1.33)  0.852 | 1.03  (0.75 1.42)  0.852 |
| Progression in superior acetabular osteophytes | 1.36  (0.76 2.43)  0.300 | 1.14  (0.65 2.00)  0.643 | 1.80  (0.97 3.34)  0.062 | 1.62  (0.91 2.89)  0.099 | 2.04  (0.79 5.22)  0.138 | 1.10  (0.37 3.30)  0.863 | 1.06  (0.93 1.19)  0.392 | 0.95  (0.84 1.07)  0.392 | 1.09  (0.89 1.34)  0.392 | 0.91  (0.74 1.12)  0.392 | 1.20  (0.79 1.80)  0.392 | 0.84  (0.55 1.26)  0.392 |
| Progression in inferior acetabular osteophytes progression~ | 1.73  (0.74 4.07)  0.208 | 1.14  (0.47 2.77)  0.768 | 2.70  (1.14 6.41)  **0.024** | 1.75  (0.71 4.32)  0.225 | 0.86  (0.10 7.48)  0.893 | 1.40  (0.29 6.65)  0.675 | 1.10  (0.91 1.34)  0.307 | 0.91  (0.75 1.10)  0.307 | 1.18  (0.86 1.62)  0.307 | 0.85  (0.62 1.16)  0.307 | 1.39  (0.74 2.64)  0.307 | 0.72  (0.38 1.36)  0.307 |
| Progression in superior femoral osteophytes | 1.15  (0.75 1.78)  0.515 | 1.04  (0.69 1.57)  0.860 | 1.05  (0.64 1.74)  0.841 | 1.05  (0.66 1.67)  0.826 | 1.24  (0.54 2.86)  0.614 | 1.28  (0.59 2.80)  0.536 | 1.02  (0.93 1.12)  0.658 | 0.98  (0.89 1.07)  0.658 | 1.04  (0.89 1.21)  0.658 | 0.97  (0.83 1.13)  0.658 | 1.07  (0.79 1.45)  0.658 | 0.93  (0.69 1.27)  0.658 |
| Progression in inferior femoral osteophytes | 1.15  (0.57 2.32)  0.702 | 1.22  (0.62 2.38)  0.568 | 0.74  (0.30 1.81)  0.512 | 0.78  (0.34 1.83)  0.574 | No observations | 0.92  (0.19 4.47)  0.919 | 1.00  (0.85 1.16)  0.950 | 1.00  (0.86 1.17)  0.950 | 0.99  (0.77 1.28)  0.950 | 1.01  (0.78 1.30)  0.950 | 0.98  (0.59 1.64)  0.950 | 1.02  (0.61 1.70)  0.950 |
| Incidence of total hip replacement | 1.04  (0.54 2.03)  0.904 | 1.20  (0.66 2.21)  0.547 | 1.15  (0.57 2.35)  0.692 | 1.09  (0.55 2.17)  0.806 | 1.25  (0.42 3.69)  0.687 | 1.35  (0.46 3.98)  0.583 | 1.00  (0.89 1.14  0.947 | 1.00  (0.88 1.13)  0.947 | 1.01  (0.82 1.24)  0.947 | 0.99  (0.81 1.22)  0.947 | 1.01  (0.67 1.53)  0.947 | 0.99  (0.65 1.49)  0.947 |
| **Pain in hip** | | | | | | | | | | | | |
| Development of frequent pain | 0.91  (0.71 1.17)  0.462 | 1.04  (0.84 1.29)  0.740 | 0.95  (0.71 1.28)  0.754 | 1.11  (0.88 1.41)  0.387 | 0.87  (0.52 1.47)  0.609 | 1.38  (0.94 2.02)  0.104 | 0.97  (0.92 1.01)  0.160 | 1.03  (0.99 1.08)  0.160 | 0.95  (0.87 1.02)  0.160 | 1.06  (0.98 1.15)  0.160 | 0.89  (0.76 1.05)  0.160 | 1.12  (0.96 1.31)  0.160 |
| Development of any pain | 0.93  (0.75 1.14)  0.479 | 0.97  (0.81 1.16)  0.725 | 0.86  (0.67 1.10)  0.227 | 0.94  (0.77 1.16)  0.582 | 0.92  (0.59 1.42)  0.692 | 0.90  (0.62 1.31)  0.591 | 0.99  (0.96 1.04)  0.779 | 1.01  (0.97 1.05)  0.779 | 0.99  (0.93 1.06)  0.779 | 1.01  (0.94 1.08)  0.779 | 0.98  (0.86 1.12)  0.779 | 1.02  (0.89 1.17)  0.779 |
| Resolution of frequent pain | 0.98  (0.76 1.27)  0.881 | 0.83  (0.66 1.05)  0.122 | 1.06  (0.79 1.42)  0.698 | 0.87  (0.67 1.14)  0.314 | 0.84  (0.48 1.45)  0.524 | 0.91  (0.58 1.43)  0.676 | 1.02  (0.97 1.07)  0.457 | 0.98  (0.93 1.03)  0.457 | 1.03  (0.95 1.12)  0.457 | 0.97  (0.89 1.05)  0.457 | 1.07  (0.90 1.26)  0.457 | 0.94  (0.80 1.11)  0.457 |
| Resolution of any pain | 0.99  (0.81 1.22)  0.946 | 1.02  (0.85 1.22)  0.816 | 1.05  (0.83 1.33)  0.697 | 1.14  (0.93 1.40)  0.201 | 0.86  (0.56 1.33)  0.495 | 1.05  (0.73 1.52)  0.774 | 0.99  (0.95 1.03)  0.541 | 1.01  (0.97 1.05)  0.541 | 0.98  (0.92 1.05)  0.541 | 1.02  (0.96 1.09)  0.541 | 0.99  (0.95 1.03)  0.541 | 1.01  (0.97 1.05)  0.541 |

Joint Space Narrowing. Adjusted for age, gender, and Body Mass Index (BMI) at baseline. ^1^ Compared to < 3% decrease or increase in BMI (stable BMI). ^2^ Compared to < 5% decrease or increase in BMI (stable BMI). ^3^ Compared to < 10% decrease or increase in BMI (stable BMI).

END OF DOCUMENT
